# Prospective association between baseline ultra-processed food consumption and future adherence to the 24-h movement guidelines in children: findings from the SENDO project

**DOI:** 10.1007/s00431-026-07287-6

**Published:** 2026-07-29

**Authors:** Nadia Paladino, Miguel Ángel Martínez-González, José Francisco López-Gil, Giuseppe Grosso, Nerea Martín-Calvo

**Affiliations:** 1 Department of Biomedical and Biotechnological Sciences, University of Catania, 95123 Catania, Italy; 2 Department of Preventive Medicine and Public Health, School of Medicine, University of Navarra, 31008 Pamplona, Spain; 3 Instituto de Investigación Sanitaria de Navarra (IdiSNA), Navarra Institute for Health Research, 31008 Pamplona, Spain; 4 Biomedical Research Network Center for Pathophysiology of Obesity and Nutrition (CIBEROBN), Carlos III Health Institute, 28029 Madrid, Spain; 5 School of Medicine, Universidad Espíritu Santo, Samborondón, Ecuador; 6 Vicerrectoría de Investigación y Postgrado, Universidad de Los Lagos, Osorno, Chile; 7 Pathophysiology of Obesity and Nutrition, Centro de Investigación Biomédica en Red, Instituto de Salud Carlos III, Madrid, Spain

**Keywords:** Diet, Physical activity, Sedentary behavior, Screen time, Sleep duration, Preschoolers

## Abstract

This study evaluates whether baseline ultra-processed food (UPF) consumption predicts failure to meet 24-h movement guidelines at 2-year follow-up in early childhood. Data from 586 participants (aged 4–5 years at baseline) from the SENDO project were analyzed. Dietary intake was assessed through a validated 149-item FFQ, completed by parents, and categorized by NOVA classification. Physical activity, screen time, and sleep time were assessed through questionnaires, based on the 24-h movement guidelines established by the Canadian Society for Exercise Physiology and the WHO. Overall, 92.0% of participants adhered to at least one guideline, 38.9% met all three, and only 8.0% met none of the recommendations. After adjusting for confounders, children in the highest UPF tertile (T3) had a rate ratio (RR) of 1.27 (95% CI 1.04–1.55; *p* = 0.021) compared with the lowest (T1). A significant linear trend was observed across UPF tertiles (*p* for trend = 0.022). Moreover, children in T3 had a greater likelihood of not meeting the recreational screen time guideline (RR 1.82, 95% CI 1.18–2.64; *p* = 0.036) than those in T1. Absolute screen time was significantly higher in T3 than T1 (1.31 ± 1.11 vs. 0.94 ± 1.18 h/day; *p* < 0.001).

*Conclusion*: This study suggests that higher UPF consumption is associated with a lower adherence to 24-h movement recommendations—specifically regarding an increased likelihood of not complying with the recreational screen time recommendation. This highlights the importance of reducing UPF intake to support healthy behaviors over time in early life.

**Table:** 

**What is Known:** • *Only a limited number of longitudinal studies have investigated the potential long-term association between dietary habits and the 24-h movement guidelines.* • *Higher consumption of ultra-processed foods (UPFs) is consistently linked to nutrient imbalances and adverse metabolic outcomes in children and adolescents.*	
**What is New:** • *This is the first study to prospectively investigate the relationship between early-life UPF intake and subsequent nonadherence to the 24-h movement guidelines in preschoolers.* • *Higher baseline UPF intake predicts a lower likelihood of meeting the movement recommendations after a 2-year follow-up, an association primarily driven by increased sedentary screen time.*

**Supplementary Information:**

The online version contains supplementary material available at https://doi.org/10.1007/s00431-026-07287-6.

## Introduction

In recent years, particularly in Western nations, dietary intakes are increasingly deficient in micronutrients and abundant in calorie-dense, ultra-processed foods (UPFs) [1]. The NOVA classification [2] categorizes food by processing level into unprocessed or minimally processed foods (MPFs), processed culinary ingredients (PCIs), processed foods (PFs), and UPFs—industrial formulations characterized by the addition of food additives [3]. Due to economic convenience, ease of use, high palatability, and extended shelf life—properties primarily driven by food additives—consumers across all age groups often favor UPFs [4, 5]. Although the long-term effects of food additives on human health remain largely unknown, research interest in their potential role in contributing to detrimental health outcomes is increasing.

The recent Lancet Series on UPFs highlighted a dramatic global rise in UPF energy share, particularly in high-income countries, where it surpasses traditional diets [6]. Moreover, higher UPF shares are consistently associated with nutrient imbalances (e.g., higher sugars and lower fiber/micronutrients) [6]. In children and adolescents globally, UPF share correlates positively with energy density and inversely with fiber [6]. Although literature is mostly adult‑focused, pediatric longitudinal work reports that higher UPF intake predicts greater body‑weight gain, fat‑mass accrual, waist‑circumference increase, and lipid abnormalities [6]. An umbrella review and meta-analysis reported significant associations between higher UPF intake and increased renal function decline, metabolic outcomes (including obesity, overweight, and diabetes), and depression in adults, as well as wheezing among children and adolescents [7]. Furthermore, UPF consumption may also negatively affect behavioral and psychological factors, including sleep quality [8], screen time [9], and physical activity (PA) [10]. Several multidimensional pathways may underlie this interplay, including behavioral clustering and parenting styles [11, 12], alongside neurobiological mechanisms involving hypothalamic neuroinflammation and reward circuitry variations [13, 14]. Nutrient imbalances typical of UPFs may also compromise circadian rhythms and sleep quality [15], underscoring the need to examine such interactions from early life due to their long-term health implications [11].

The need for an integrated approach led to the development of “24-Hour Movement Guidelines” by the Canadian Society for Exercise Physiology, providing age-specific recommendations for sleep duration, physical activity, and recreational screen time within a 24-h period [11]. Although dietary habits and movement behaviors are interrelated [16], few articles have focused on their long-term associations [17]. A recent systematic review revealed that children and adolescents adhering to all three 24-h movement recommendations had higher intakes of fruit, vegetables, nuts, and fish and a lower consumption of sweets and pastries, compared to non-adherent peers [17].

While cross-sectional studies examined isolated lifestyle factors in relation to UPF consumption [10, 18, 19], no prior research has investigated the longitudinal relationship between UPF intake and meeting 24-h movement recommendations among preschoolers. To fill this gap, this study evaluates the longitudinal association between UPF consumption at 4–5 years of age and meeting the 24-h movement guidelines after 2 years of follow-up within the SENDO project.

## Materials and methods

### Study population

**Fig. 1:**
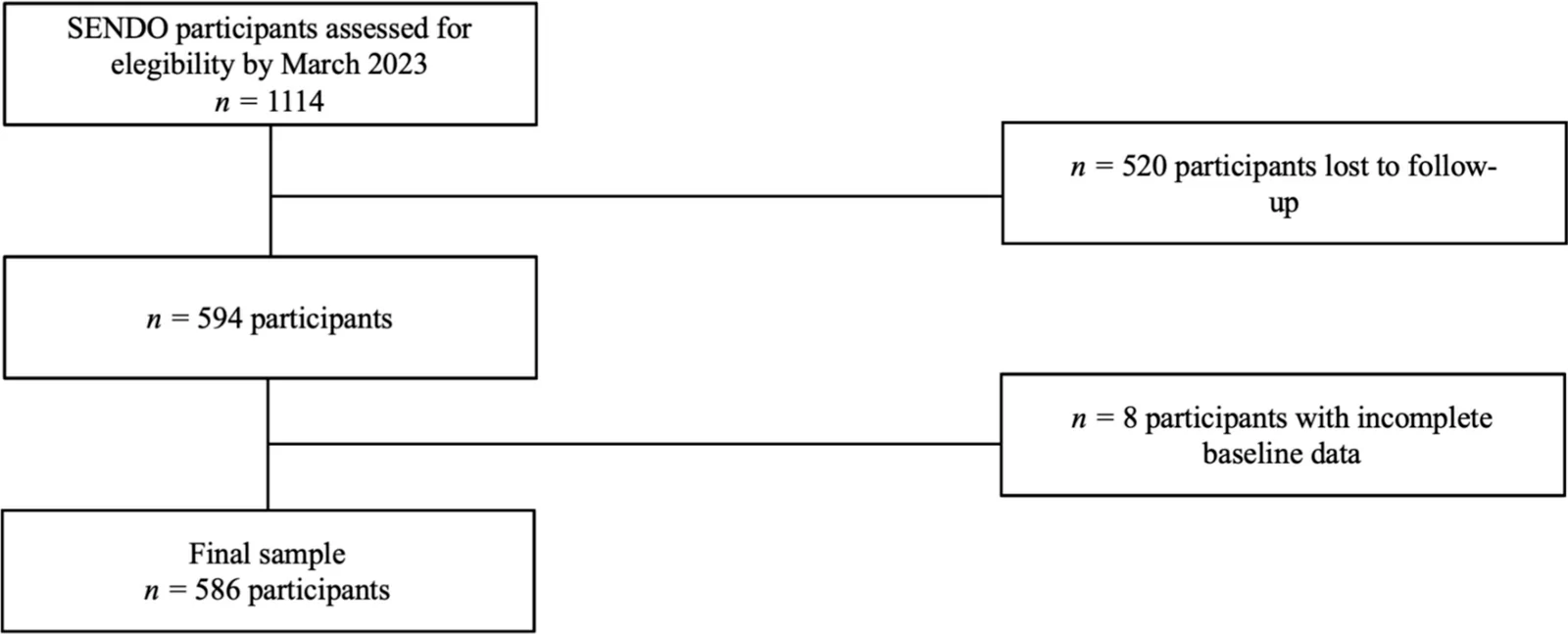
Flowchart reporting the SENDO participants included in the analyses

The *Seguimiento del Niño para un Desarrollo Óptimo* (SENDO) project is an ongoing prospective Spanish cohort designed to assess the potential relationships of diet and lifestyle factors with children’s health. Eligible children were aged 4–5 years and resided in Spain; the sole exclusion criterion was the lack of internet access to complete the questionnaires. Participants were enrolled via pediatricians at primary healthcare centers or through schools. Parents complete self-administered online questionnaires, which are updated annually. Participant recruitment started in January 2015 (Q0), and for the present study, an initial subsample of the cohort was selected (*n* = 1114) by including subjects recruited before March 2023 to allow sufficient time for a 2-year follow-up (Q2). A total of 520 (46.68%) individuals were excluded because they were lost to follow-up. No significant differences in baseline UPF intake or 24-h recommendation adherence existed between retained and lost-to-follow-up participants (Supplementary Materials, Supplementary Table 1). Eight participants were subsequently excluded because of incomplete baseline data (Q0), leaving a final analytical sample of 586 participants. The participants' selection process is detailed in Fig. 1.

At baseline (Q0), data on sociodemographic characteristics, anthropometrics, dietary habits, lifestyles, and physical activity were collected. Provided information is entered electronically and stored within a secure online database. The 2-year follow-up questionnaire (Q2) updates and extends the baseline data.

### Ethical approval

SENDO adheres to the ethical guidelines outlined in the Helsinki Declaration concerning ethical principles in human research. The project’s protocol received approval from the Ethical Committee for Clinical Research of Navarra (Pyto 2016/122). Parents provided written informed consent via mail prior to SENDO project enrollment.

### Assessment of 24‐h movement recommendations

The 24-h movement recommendations were defined according to Tremblay and colleagues [20] and the World Health Organization (WHO) [21]. Physical activity was evaluated via a questionnaire asking about 17 moderate-to-vigorous activities and 10 response options, ranging from “never” to “over 10 h per week.” The Spanish version of the questionnaire underwent initial validation within the *Seguimiento Universidad de Navarra* (SUN) study [22]. In accordance with the WHO criteria [21], for children aged < 5 years, adherence was defined as a minimum of 180 min/day of PA, including ≥ 60 min/day of intense/vigorous play. For children aged ≥ 5 years, adherence required accumulating ≥ 60 min/day of MVPA. Screen time and sleep duration were assessed across both weekdays and weekends. Children aged < 5 years were adherent if recreational screen time was ≤ 1 h/day and sleep duration was 10–13 h/day. For children aged ≥ 5 years, thresholds were set to a maximum of 2 h/day for screen time and 9–11 h for sleep. Participants meeting the respective criteria for all three domains were classified as fully adhering to the recommendations. Further details regarding the operationalization and methodology of the 24-h movement framework assessment are provided in Supplementary Materials.

### Assessment of UPF consumption

Dietary information was collected via a previously validated 149-item semiquantitative food frequency questionnaire (FFQ) [23]. For each item, parents reported their child’s frequency of consumption by choosing across nine categories, from “never or almost never” to “ ≥ 6 times per day.” Nutrient intake per item was calculated by multiplying reported consumption frequency by the edible portion and item composition from updated Spanish food composition tables [24]. Food items were classified according to the NOVA system [5] following a post-hoc disaggregation protocol (Supplementary Materials). The percentage of the total energy intake (TEI) that came from each NOVA group was calculated as (energy contribution of each group/TEI) × 100.

### Assessment of covariates

Sociodemographic, lifestyle, and dietary information (including sex, weight, height, maternal education, and siblings) were collected via questionnaires administered to parents at baseline (Q0). Maternal and participant ages were calculated as the difference between the questionnaire receipt date and their respective birth dates. The body mass index (BMI) z-scores and body mass status were defined based on age- and sex-specific cutoffs from the International Obesity Task Force [25]. Supporting the reliability of this proxy reporting, a previous study reported high concordance between parental data and direct measurements [26].

### Statistical analysis

Participants were classified into tertiles (T) based on baseline UPF energy intake derived from the FFQs completed by parents. Numbers (percentages) for categorical variables and means (standard deviations) for quantitative variables were used, following verification of their symmetrical distribution. Additionally, differences in absolute recreational screen time (expressed as hours/day and including television, computer, and tablet use) across UPF consumption tertiles were evaluated using the Kruskal–Wallis test. By allocating the median UPF consumption to each tertile and assuming that this variable is continuous, linear trend tests were performed across tertiles. Prospective associations between basal UPF consumption, considered an independent variable, and the number of unmet 24-h movement recommendations after 2 years of follow-up, as a dependent variable, were assessed via Poisson generalized estimating equation (GEE) models, which accounted for the intracluster correlation between siblings. Rate ratios (RRs) and their 95% CIs were calculated considering the lowest tertile as the reference category (T1). All associations were examined by considering UPF consumption both as a categorical variable (in tertiles) and as a trend variable for the linear trend test. To account for potential confounders, two progressively adjusted models were used: Model 1 was adjusted for age, sex, recruitment date, and the number of 24-h movement recommendations unmet at baseline; model 2 was additionally adjusted for participants’ body mass status, maternal higher education, and the number of siblings. Full details on adjustment categories and missing data handling are provided in Supplementary Materials. Chi-square (chi^2^) tests and Phi (φ) coefficients were computed to assess the internal correlations and behavioral clustering among the nonadherence to the three 24-h movement guidelines at follow-up. Following this, an additional prospective analysis assessing nonadherence to each specific 24-h movement guideline was conducted. Given the binary nature of the individual components, initial GEE models used a binomial distribution with a logit link, yielding odds ratios (ORs) converted to RRs using Zhang and Yu’s formula [27] to ensure accurate interpretation. Associations were adjusted for baseline age, sex, recruitment date, body mass status, maternal higher education, and number of siblings. Each recommendation was further adjusted for baseline nonadherence to the respective guideline. Additionally, to enhance clinical interpretability, post-estimation marginal effects were used to compute adjusted predicted risks and adjusted absolute probabilities after the fully adjusted models. All *p* values are two-tailed. Statistical significance was set at the conventional cutoff point of *p* < 0.05. Analyses were carried out via Stata version 19.5 (Stata Corporation).

## Results

A total of 586 participants were included in the present study. Table 1 shows participant and parental characteristics across UPF consumption tertiles. Both maternal age and the proportion of mothers with high educational attainment were similar across tertiles. Conversely, participants in the higher UPF tertiles tended to be marginally older. Furthermore, the proportion of females differed between the tertiles, and variations were observed for the proportion of children with a birthweight ≥ 3500 g. Regarding the number of siblings, the proportion of participants with 3 or more siblings varied across tertiles. In addition, the distribution of caloric intake differed slightly between participants in the highest UPF tertile (T3) and those in the lowest tertile (T1). The mean contribution of UPFs to total daily energy intake was 26.11% in T1, 37.16% in T2, and 47.88% in T3. Finally, although the mean Z scores of BMI ranged from − 0.03 in the lowest UPF tertile to 0.12 in the highest UPF tertile, the distributions across UPF categories of consumption remained nearly identical.

**Table 1:** Baseline characteristics of participants (*n* = 586) in the SENDO project and their families by tertiles of ultra-processed food consumption enrolled between January 2015 and March 2023. The numbers are the means (SDs) or *n* (%)

	T1 (*n* = 196)	T2 (*n *= 195)	T3 (*n* = 195)
*Parental characteristics*			
Maternal age (years)	39.78 (4.03)	40.09 (3.97)	40.14 (3.99)
Maternal high education, *n* (%)	159 (27.13)	172 (29.35)	151 (25.77)
*Children characteristics*			
Sex (female), *n* (%)	106 (18.09)	92 (15.70)	91 (15.53)
Age (years)	4.73 (0.72)	4.92 (0.82)	4.92 (0.77)
Birthweight, *n* (%)			
< 2500 g	19 (3.24)	15 (2.56)	20 (3.41)
2500–3500 g	119 (20.31)	116 (19.80)	102 (17.41)
≥ 3500 g	58 (9.90)	64 (10.92)	73 (12.46)
Weight (kg)	18.78 (3.43)	19.36 (3.32)	19.65 (3.16)
Height (cm)	109 (7.09)	111 (6.91)	112 (7.02)
Z score of the BMI^a^	− 0.03 (1.11)	0.08 (1.15)	0.12 (1.08)
Body mass status^a^, *n* (%)			
Underweight	33 (5.63)	29 (4.95)	25 (4.27)
Normal weight	142 (24.23)	142 (24.23)	148 (25.26)
Overweight/obesity	21 (3.58)	24 (4.10)	22 (3.75)
Total energy intake (kcal/day)	1987 (520)	2015 (497)	2087 (532)
UPF energy intake (% of total kcal)	26.11 (5.08)	37.16 (2.33)	47.88 (5.43)
Number of siblings, *n* (%)			
None	37 (6.31)	21 (3.58)	27 (4.61)
One sibling	118 (20.14)	116 (19.80)	107 (18.26)
Two siblings	35 (5.97)	31 (5.29)	37 (6.31)
Three or more siblings	6 (1.02)	27 (4.61)	24 (4.10)

*BMI* body mass index, *SD* standard deviation

^a^According to age- and sex-specific cutoff points from the International Obesity Task Force [25]

**Fig. 2:**
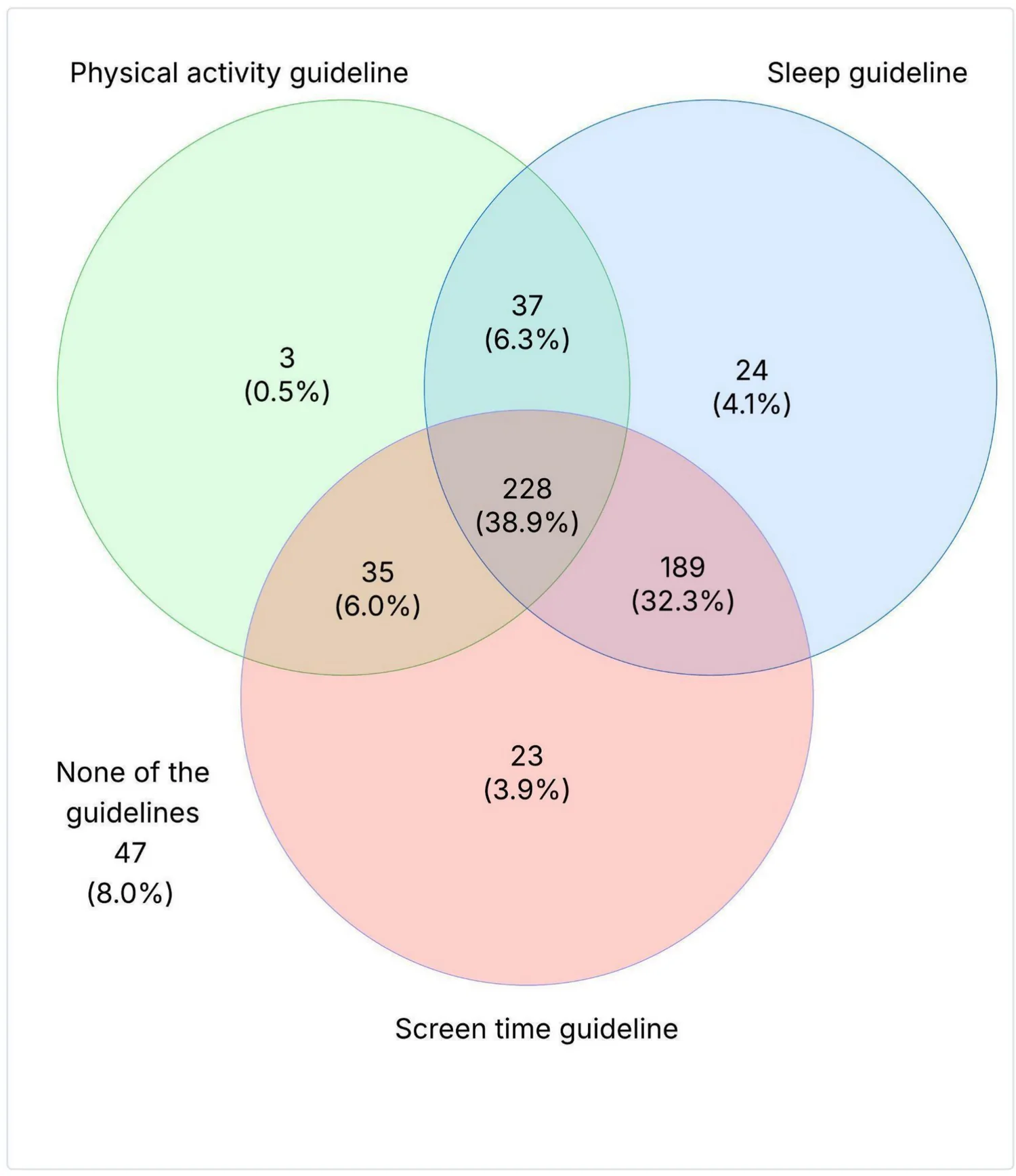
Venn diagram of the distribution of participants (*n* = 586) adhering to each 24-h recommendation at follow-up. Numbers and percentages represent the children meeting the 24-h movement guidelines. The areas of intersection indicate the percentage of participants who simultaneously met two or three recommendations (i.e., the intersection of “Physical activity” and “Sleep” (6.3%) represents the proportion of children meeting these two guidelines but not the “Screen time” one). The central intersection (38.9%) represents the proportion of participants meeting all three guidelines

Figure 2 shows the distribution of children meeting each 24-h movement guideline at follow-up. Specifically, most participants adhered to at least one guideline (92.0%, *n* = 539). A total of 228 participants (38.9%) met all three guidelines simultaneously; only 47 children (8.0%) met none of the recommendations. The most common combination of adherence was sleep and screen time only (*n* = 189, 32.3%), followed by PA and sleep (*n* = 37, 6.3%) and PA and screen time (*n* = 35, 6.0%). For individual recommendations, 24 children (4.1%) met the sleep guideline only, whereas 23 participants (3.9%) met the screen time recommendation exclusively. Only 3 participants (0.5%) adhered to the PA guideline exclusively.

The internal correlation analysis among the three 24-h movement guidelines at follow-up revealed a significant and mutual interdependence among all components (all *p* < 0.001). Specifically, nonadherence to the PA guideline was positively associated with noncompliance with both the screen time (chi^2^ = 13.47, *p* < 0.001, φ = 0.15) and the sleep duration guideline (chi^2^ = 14.47, *p* < 0.001, φ = 0.16). Similarly, a strong correlation was observed between the nonadherence to screen time and sleep duration guidelines (chi^2^ = 64.52, *p* < 0.001, φ = 0.33).

Table 2 shows the prospective association between baseline UPF consumption and the number of unmet 24-h movement recommendations after 2 years of follow-up.

**Table 2:** Rate ratios (RRs) and 95% confidence intervals (CIs) for the prospective association between baseline ultra-processed food consumption and noncompliance with the 24-h movement recommendations

Ultra-processed food consumption at baseline			
*Unadjusted*	RR (95% CI)	*p *value	*p *for trend
T1	1.00 (Ref.)		
T2	1.05 (0.85, 1.29)	0.679	
T3	1.34 (1.10, 1.64)	**0.003**	**0.001**
*Model 1*			
T1	1.00 (Ref.)		
T2	1.05 (0.85, 1.29)	0.676	
T3	1.27 (1.04, 1.55)	**0.019**	**0.020**
*Model 2*			
T1	1.00 (Ref.)		
T2	1.06 (0.86, 1.31)	0.576	
T3	1.27 (1.04, 1.55)	**0.021**	**0.022**

Model 1 is adjusted for age at baseline, sex, date of recruitment, and the number of 24-h movement guidelines not met at baseline

Model 2 was further adjusted for body mass status (underweight, normal weight, overweight/obesity), maternal education level (university degree, master’s degree or doctorate), and number of siblings

In the unadjusted model, participants in the highest tertile of UPF consumption reported a higher rate of unmet 24-h movement recommendations (RR 1.34, 95% CI 1.10–1.63; *p* = 0.004) compared with children in the lowest tertile. Furthermore, the association remained statistically significant even after adjustment for potential confounding factors. In the fully adjusted model, participants in T3 had a greater rate of unmet 24-h movement recommendations (RR 1.27, 95% CI 1.04–1.55; *p* = 0.021) than did their peers at T1. A significant linear trend was observed across the tertiles of UPF consumption (*p* for trend = 0.022), with adjusted absolute probabilities of non-adherence ranging from 0.77 (95% CI 0.65–0.89) in the lowest tertile to 0.98 (95% CI 0.85–1.10) in the highest tertile.

**Fig. 3:**
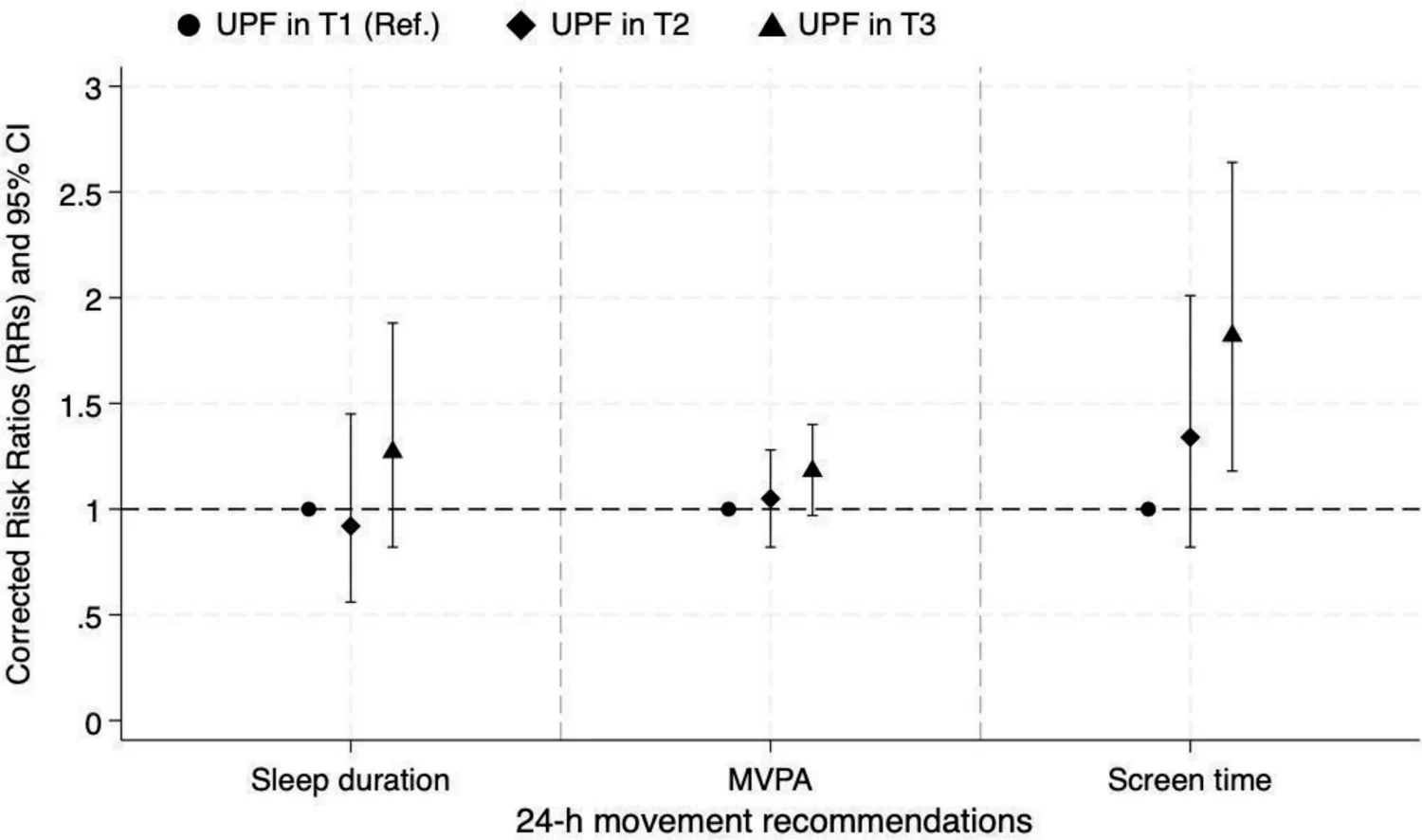
Two-way diagram reporting the prospective association between baseline ultra-processed food consumption and future nonadherence to each specific item of the 24-h movement recommendations. ^a^Corrected risk ratios (RRs) were obtained via the formula proposed by Zhang and Yu [27] to convert odds ratios (ORs) to risk ratios (RRs). All associations were adjusted for age at baseline, sex, date of recruitment, body mass status (underweight, normal weight, overweight/obesity), maternal education level (university degree, master’s degree, or doctorate), and number of siblings. Each recommendation (sleep duration, time spent on moderate-to-vigorous physical activity, and recreational screen time) was further adjusted for baseline nonadherence to the respective guidelines. Abbreviations: MVPA (moderate-to-vigorous physical activity); UPF (ultra-processed food)

Nonadherence to each specific 24-h movement recommendation and baseline UPF consumption is graphically presented in Fig. 3. The results showed that children with higher baseline UPF consumption (T3) had a greater risk of exceeding the recreational screen time guideline (RR 1.82, 95% CI 1.18–2.64; *p* = 0.036) than did those in the lowest UPF consumption tertile (T1). In particular, the adjusted absolute probabilities of non-adherence to screen time guidelines ranged from 0.15 (95% CI 0.10–0.20) in T1 to 0.23 (95% CI 0.18–0.29) in T3. Specifically, the absolute time spent on recreational screens was significantly higher in T3 compared to T1 (mean ± SD 1.31 ± 1.11 h/day vs. 0.94 ± 1.18 h/day, respectively; *p* < 0.001). Regarding adherence to the specific recreational screen time recommendation within the 24-h movement guidelines (≤ 2 h/day for children aged ≥ 5 years), the upper distribution within the highest UPF tertile reached 2.42 h/day. This indicates that the segment of children within T3 who did not adhere to the recommendations exceeded the daily threshold by up to 0.42 h/day (approximately 25 min/day), whereas their peers in T1 exceeded it by only 0.12 h/day (approximately 7 min/day).

## Discussion

The present study prospectively evaluated the association between UPF consumption at 4–5 years of age and subsequent nonadherence to the 24-h movement guidelines after 2 years of follow-up in the SENDO cohort. Our findings suggest that children with higher UPF consumption were less likely to meet 24-h movement guidelines, an association predominantly driven by an increased likelihood of noncompliance with the recreational screen time recommendation. Interestingly, while recent prospective evidence in adults linked UPF intake to physical inactivity [28], our findings in early childhood highlight a primary association with screen time. This aligns with a recent systematic review confirming a strong cross-sectional co-occurrence between unfavorable dietary patterns—particularly higher UPF consumption—and compromised movement behaviors, which often cluster as “High Sedentary Behavior, High UPF, Low Sleep” [29]. Particularly, a cross-sectional SENDO study revealed that children who met all three 24-h movement recommendations consumed more fruits, vegetables, and nuts than those who did not meet the recommendations [30]. Moreover, a recent systematic review highlighted that children adhering to all three guidelines tended to have healthier dietary patterns [17]. In our sample, 92.0% of participants adhered to at least one guideline, 38.9% met all three, and 8.0% met none. By comparison, recent pediatric studies report adherence rates to all three guidelines ranging between 10.4 and 19.0% among children [31, 32]. Furthermore, our longitudinal findings suggest an additional and previously unreported direction of the association: poor dietary habits, such as higher UPF consumption, may precede the subsequent adoption of a worse overall lifestyle. The prospective nature of the relationship is supported by a longitudinal study of 202 Chinese children, which demonstrated that baseline adherence to all three movement guidelines was associated with a smaller increase in waist-to-height ratio, while meeting both screen time and sleep guidelines was linked to lower adiposity change [33]. Nevertheless, when interpreting our results, the possibility of reverse causation warrants consideration, as children with increased screen time are heavily exposed to targeted advertising and mindless snacking on UPFs [34]. This complements, rather than excludes, the traditional causal pathway. Furthermore, individual clinical relevance differs from public health importance [35]; despite modest prospective effect sizes, widespread UPF exposure among children yields substantial population-level implications [36]. In epidemiological research, a modest individual effect size may translate into a substantial cumulative burden across a population over time [35]. Given the low pediatric adherence rates to movement guidelines [31] and their decline with age [32], early childhood represents a critical window for intervention before these subtle, compounding behavioral shifts consolidate [37]. Particularly, although screen time differed by only 18 min/day between extreme UPF tertiles, this translates to roughly 2 additional hours/week during a critical developmental period. Importantly, children in the highest UPF tertile exceeded the 2-h threshold by 25 min/day, compared to only 7 min in the lowest tertile, indicating that higher UPF consumption systematically shifts children further above recommendations. Furthermore, this temporal overlap should be contextualized within behavioral and environmental patterns, as highly palatable UPFs are frequently consumed as convenient snacks during passive screen activities in early childhood [38]; thus, this 18-min window may realistically reflect a single distracted eating episode or mealtime routine in front of a screen, suggesting these behaviors are contextually linked rather than independent.

Our finding that the screen time recommendation was the most likely to be unmet is supported by the literature on behavioral clustering. A cross-sectional study included in the systematic review by Zeng and colleagues [17] revealed that the screen time recommendation has been identified as the individual guideline most strongly associated with dietary patterns [39], thereby substantiating its role as the primary statistical driver within our prospective association.

One reason for this prospective relationship may be the clustering of unhealthy dietary and movement behaviors [40]. The concept of clustering is based on the premise that health behaviors co-occur and influence each other within the same individual [41]. Thus, a specific profile may be defined when one unhealthy characteristic (i.e., higher UPF intake) increases the probability of engaging in others (i.e., excessive screen time) [41]. Consistently, a significant mutual interdependence among the noncompliance with all three movement components at follow-up was confirmed, specifically linking excessive screen time and insufficient sleep. However, higher baseline UPF intake was associated only with future nonadherence to screen time guidelines, which suggests that in early childhood, sedentary screen behavior may serve as the primary behavioral interface through which baseline dietary quality impacts the 24-h movement framework over time. Crucially, these clustered behaviors may synergistically amplify health risks beyond their simple additive effects [41].

The longitudinal associations may be explained by cultural and educational factors linked to the four different parenting styles, which balance responsiveness and demandingness [42]. The authoritative style optimizes emotional regulation, whereas the authoritarian focuses on obedience. The permissive and neglectful styles reflect difficulties in setting boundaries or lack of involvement, respectively [42]. Although parenting styles were not directly measured in the present study, enforcing boundaries around diet and screen time requires substantial parental energy and supportive environments [12]. However, structural barriers like time scarcity and environmental pressures often drive a shift toward permissive parenting [43], indirectly reinforcing the clustering of unhealthy dietary and sedentary behaviors.

Another potential explanation may be the link between appetite control and UPF overconsumption. Satiety is regulated by a complex network of peripheral hormones (i.e., insulin, cholecystokinin, and leptin, which promote fullness, whereas the orexigenic hormone ghrelin falls) and central hypothalamic circuits, whose hormones converge on the arcuate nucleus [13]. As energy-dense foods with increased palatability [5], UPFs—typical of high-fat diets (HFDs)—may induce neuroinflammation within satiety centers (i.e., in the hypothalamus) [14]. Animal studies suggest that HFD exposure drives neuronal mitochondrial dysfunction, leading to leptin and insulin resistance, which alters innate hunger-satiety signals and exacerbates this vicious cycle [14].

Moreover, brain regions involved in the reward are highly responsive to food stimuli with high appetitive value [44], with UPF consumption potentially inducing a powerful dopamine release [45]. This neural circuitry is activated similarly by both addictive drugs and UPFs, leading to an escalation in intake and making it more difficult to reduce consumption [46]. In fact, the transient reward sensation derived from UPFs reinforces this behavior, compelling individuals to repeat the experience [47]. Although evidence stems primarily from animal models, emerging human studies suggest that UPF consumption may trigger tolerance and withdrawal symptoms [48, 49]. Palatable UPFs may initially cause a rapid release of dopamine, but chronic overconsumption downregulates striatal dopamine D₂ receptors, driving larger intakes to achieve the same reward [14, 50]. However, this evidence remains nuanced, largely due to UPF heterogeneity [51]. While food addiction applies well to hyper-palatable UPFs rich in fats and sugars, other ultra-processed items like specific commercial breads or enriched cereals may be higher in fiber [52], suggesting that reward-system activation is not a uniform response to all UPFs. Moreover, children exhibit greater reward sensitivity [52] alongside ongoing prefrontal cortex development [53], showing a higher propensity for risk-taking factors, with constant UPF seeking directly impacting how their reward system processes stimuli [54]. Furthermore, early UPF exposure may induce long-term functional changes in neural reward systems that strengthen the association with simultaneous passive behaviors [47]. This clustering is perpetuated by a vicious cycle: sedentary behaviors have been consistently associated with a greater intake of highly palatable, calorie-dense foods [55]. Children and adolescents are constantly exposed to advertisements for foods high in fat and sugar while engaging in sedentary activities, inducing them to overeat [47]. The phenomenon of “mindless eating” consists of increased food consumption while distracted, mainly reported among youth who spend excessive time in front of screens [56, 57]. Furthermore, recent human trials reveal that UPFs are typically characterized by a soft texture and low structural complexity that require minimal chewing, substantially increasing oral processing speed and driving larger dietary intakes [58]. This mechanical property of UPF matrices may act synergistically with screen-related distraction, further weakening natural appetite control mechanisms.

This study is subject to several methodological limitations. First, dietary assessment relies on FFQs, which are susceptible to measurement error and recall bias [59]. Furthermore, both dietary and lifestyle habits rely entirely on parental reporting, potentially introducing social desirability bias and overestimating compliance [60]. Future research employing device-based measurements for physical activity, sedentary behavior, and sleep duration is recommended to increase objective accuracy. Second, the specific FFQ used was not designed according to the NOVA classification [23], as it lacked detailed information regarding ingredients or food additives. Moreover, although the NOVA classification is the most widely used method for assessing food processing levels [61], it is not without criticism. The most significant issue is its focus solely on the level of processing rather than the nutritional profile [51]; however, this distinction is a recognized challenge common to other food categorizations, such as the International Agency for Research on Cancer-European Prospective Investigation into Cancer and Nutrition (IARC-EPIC) [62]. Furthermore, the demonstrated association between higher UPF consumption and detrimental health outcomes in both adults and children [63, 64] justifies its utility. The SENDO cohort is based on a volunteer sample and may not be fully representative of the general Spanish population, i.e., with a notable overrepresentation of caregivers reporting higher educational attainment. Nevertheless, this demographic profile provides a methodological advantage, as the reduced heterogeneity minimizes residual confounding by education and socioeconomic status, thereby increasing internal validity [65]. Furthermore, the possibility of residual confounding cannot be completely dismissed. Specifically, family-level determinants—such as parenting practices, household screen rules, parental lifestyles, and socioeconomic characteristics—were not collected within the SENDO project, potentially confounding the observed associations. Another methodological limitation is that UPF intake was assessed only at baseline. Dietary habits may change during childhood, and the possibility of exposure misclassification over time cannot be excluded. Nevertheless, the primary focus was to evaluate how early-life baseline UPF exposure may influence future behavioral trajectories. Finally, we acknowledge a high proportion of participants lost to follow-up; however, for any selection bias to explain our results, study inclusion would need to be associated with UPF consumption or with meeting the 24-h movement recommendations, which cannot be supported by the available data.

Despite these limitations, the study has several important strengths. First, the prospective design establishes the antecedence of the exposure over the outcome, reducing the risk of reverse causality common in cross-sectional designs. Moreover, the focus on early childhood is critical, providing evidence for the antecedent role of baseline UPF consumption in establishing later noncompliant lifestyle patterns during a key developmental window. Second, the models were progressively adjusted for critical sociodemographic factors and for the baseline status of all outcome components (i.e., baseline number of unmet movement guidelines), ensuring that the observed prospective associations are independent of existing movement behaviors at baseline. Furthermore, the use of GEEs properly handles the nonindependence of repeated measures collected from the same participants over time, enhancing the robustness of the findings. Third, the study gains significant strength from using the 24-h movement framework to integrate physical activity, sedentary time, and sleep, aligning the research with contemporary public health paradigms that emphasize the interdependence of these behaviors.

In conclusion, this study provides novel prospective evidence supporting a significant relationship between UPF consumption at 4–5 years of age and subsequent nonadherence to the 24-h movement recommendations among children from the SENDO project. The association is predominantly driven by an increased likelihood of noncompliance with the recreational screen-time recommendation, which highlights the synergistic clustering between poor dietary habits and sedentary behavior during early childhood. The prospective design of this study is crucial, as it suggests that early-life dietary choices may precede and contribute to the establishment of future detrimental lifestyle patterns. This underscores the importance of adopting an integrated 24-h perspective in public health, recognizing that food choices, specifically a higher consumption of UPFs, are inherently intertwined with movement behaviors. Consequently, future public health initiatives may benefit from combining the 24-h movement recommendations with dietary guidelines.

## Supplementary Information

Below is the link to the electronic supplementary material.

ESM 1 (DOCX 3.80 MB)

## Data Availability

Data will be available upon a reasonable request.

## Abbreviations

BMI: Body mass index
CI: Confidence interval
FFQ: Food-frequency questionnaire
HFDs: High-fat diets
HFCS: High-fructose corn syrup
METs: Metabolic equivalents of task
MVPA: Moderate-to-vigorous physical activity
MPFs: Minimally processed foods
PA: Physical activity
PCIs: Processed culinary ingredients
RRs: Rate ratios
SD: Standard deviation
SENDO: Seguimiento del Niño para un Desarrollo Óptimo
T1-T2-T3: First, second and third tertiles
UPF: Ultra-processed food
