# Supplementary material for: Prospective association between baseline ultra-processed food consumption and future adherence to the 24-h movement guidelines in children: findings from the SENDO project
Source: Eur J Pediatr. 2026 Jul 29;185(8):621. doi: 10.1007/s00431-026-07287-6 (PMC13421281; doi:10.1007/s00431-026-07287-6)
Supplement: Supplementary file 1 — (DOCX 3.80 MB) [file 431_2026_7287_MOESM1_ESM.docx]

Supplementary Materials

*European Journal of Pediatrics*

**Prospective association between baseline ultra-processed food consumption and future adherence to the 24-h movement guidelines in children: findings from the SENDO project**

Nadia Paladino, Miguel Ángel Martínez-González, José Francisco López-Gil, Giuseppe Grosso, Nerea Martín-Calvo^a^

**^a^Corresponding author:** Dr. Nerea Martín-Calvo, Department of Preventive Medicine and Public Health, School of Medicine, University of Navarra, 31008 Pamplona, Spain. E-mail: [nmartincalvo@unav.es](mailto:nmartincalvo@unav.es)

**Supplementary Table 1** Differences in baseline ultra-processed food (UPF) intake and the number of 24-h movement recommendations met among included and lost-to-follow-up participants

|  | **Included**  **(n = 586)** | **Lost to follow-up**  **(n = 414)^a^** | ***p value*** |
| --- | --- | --- | --- |
| Baseline UPF intake, mean (SD) | 37.03 (9.97) | 36.79 (10.11) | 0.71 |
| Baseline UPF intake, n (%) |  |  | 0.78 |
| T1 | 199 (19.90) | 135 (13.50) |  |
| T2 | 190 (19.00) | 143 (14.30) |  |
| T3 | 197 (19.70) | 136 (13.60) |  |
| Number of 24-h movement  guidelines met at baseline, mean (SD) | 1.43 (0.97) | 1.43 (1.03) | 0.98 |
| Number of 24-h movement  guidelines met at baseline, n (%) |  |  | 0.20 |
| None | 100 (10.00) | 84 (8.40) |  |
| One recommendation | 237 (23.70) | 154 (15.40) |  |
| Two recommendations | 147 (14.70) | 90 (9.00) |  |
| Three recommendations | 102 (10.20) | 86 (8.60) |  |

Quantitative data are expressed as the means and standard deviations (SDs); qualitative data are expressed as numbers (n) and percentages.

^a^A total of 114 participants were previously excluded because they lacked baseline UPF data.

**Assessment of 24‐h movement recommendations (dependent variable)**

To calculate the metabolic equivalents of task (METs) for each activity, the MET value assigned to each activity was multiplied by the weekly frequency of engagement, adjusting for the number of months during which the activity was sustained (1). Activities exceeding 5 METs were considered vigorous, while those with a value ≤5 METs were considered moderate. To determine adherence to the guidelines, questionnaire measures were operationalized into daily metrics based on these specific intensity domains. Parent-reported weekly hours dedicated specifically to moderate physical activity (≤5 METs) and intense/vigorous play (>5 METs) were converted into daily minutes (calculated as: hours × 60/7). To average both screen time (including activities such as watching TV, using a computer, or playing video games) and sleep time, the following formula was used: [(average time on weekdays × 5) + (average time on weekends × 2)] divided by 7.

**Assessment of UPF consumption (independent variable)**

The NOVA classification proposed by Monteiro and colleagues (5) categorizes each item into four groups on the basis of its level of processing: (i) unprocessed or minimally processed foods (consisting of natural foods altered by processes such as drying, boiling, or freezing without the addition of any substance); (ii) processed culinary ingredients (foods not eaten as such but used as cooking ingredients); (iii) processed foods (simple products manufactured by adding Group 2 ingredients to Group 1 foods to alter durability or palatability); and (iv) ultra-processed foods (industrial formulations typically containing five or more ingredients, characterized by the inclusion of food additives). Rather than allocating whole composite food items directly to a single NOVA category, these items were broken down into their constituent recipe fractions. In the absence of brand names or specific food additive lists, the classification of components was guided by the commercial nature of the parent food item in the marketplace. For items identified as commercially manufactured (e.g., packaged baked foods, breakfast cereals, or ready-to-eat meals), ingredients characteristically associated with industrial processing were conservatively allocated to the ultra-processed category (NOVA 4). All other constituent ingredients within the recipe were systematically assigned to Group 1, 2, or 3 based strictly on their individual degree of processing as defined by the NOVA criteria. For each disaggregated fraction, grams and energy contributions from carbohydrates, proteins, and lipids were individually calculated and assigned to their corresponding processing level. Total energy intake for each NOVA group was subsequently computed by summing the specific caloric contributions of carbohydrates, proteins, and lipids assigned to that group.

**Missing data management and adjustment categories**

Multivariable GEE models were fitted using progressive adjustment levels to control for potential confounders. Model 1 was adjusted for baseline age (continuous), sex (male/female), recruitment period (categorized as 2015–2016, 2017–2018, 2019–2020, 2021–2022, or 2023), and the baseline number of unmet 24-h movement recommendations (0, 1, 2, or 3). Model 2 (fully adjusted) was additionally adjusted for participants' body mass status (underweight, normal weight, or overweight/obesity), maternal higher education level (university degree, master's degree, or doctorate), and the number of siblings (none, 1 sibling, 2 siblings, or 3 or more siblings).

Missing data were minimal across the analytical sample. For the number of siblings covariate, missing values were handled via logical imputation, assigning them to the “3 or more siblings” category based on baseline characteristics. For all other covariates, a complete-case analysis approach was maintained. A minimal missingness (0.5%) was observed only for the date of recruitment variable (n = 3) due to missing exact enrollment dates in pilot participants, who were nonetheless all recruited in 2015. As the precise dates were unavailable, no logical imputation could be performed for this variable, leading to the automatic exclusion of these participants by the statistical software; consequently, the fully adjusted model was computed on 583 participants. No statistical imputation procedures were performed.

**Reference**

1[. Ainsworth BE, Haskell WL, Herrmann SD, Meckes N, Bassett DR, Tudor-Locke C, et al. 2011 Compendium of Physical Activities: a second update of codes and MET values. Med Sci Sports Exerc. 2011 Aug;43(8):1575–81.](https://sciwheel.com/work/bibliography/3299800)
